# Assessment of malformations, variations and diameters of vessels forming the circle of Willis – An autopsy study in a non-cerebrovascular cohort

**DOI:** 10.1515/tnsci-2022-0253

**Published:** 2022-11-08

**Authors:** László Orosz, Zoltán Gyöngyösi, Zsolt Susán, Péter Siró, Arjan Willem Hoksbergen, László Csiba, Béla Fülesdi

**Affiliations:** Department of Surgery, University of Debrecen, Debrecen, Hungary; Department of Anesthesiology and Intensive Care, University of Debrecen, H-4032, Nagyerdei krt. 98, Debrecen, Hungary; Department of Vascular Surgery, Academic Medical Center, Amsterdam, The Netherlands; Department of Neurology, Faculty of Medicine, University of Debrecen, H-4032, Nagyerdei krt. 98, Debrecen, Hungary

**Keywords:** circle of Willis, autopsy, diameter, configuration

## Abstract

**Background a purpose:**

The collateral capacity of the circle of Willis (CoW) may play an important role in the development of ischemic strokes. The occurrence of classical polygon shows wide geographical variations and morphological data on diameters of the Willisian collaterals are scarce. We aimed to assess CoW variations and vessel diameters in a Central European cohort.

**Subjects and methods:**

CoWs were removed during routine autopsy. The morphological pattern of the circles was recorded. The prepared circles were then put between two glass plates and tightly compressed. The length of the vessels and half of the circumference were measured under a light microscope enabling measurement with an approximation of 0.1 mm. Vessel diameters were calculated from vessel circumference.

**Results:**

A total of 110 circles were analysed. Incomplete circles (missing one or two segments of CoW) were found in 25 cases (22.7%). Any forms of anatomical variations were detected in 14 cases (12.7%). When applying the <1 mm diameter threshold for analysis, 36 anterior communicating arteries (32.7%), 53 right posterior communicating arteries (48.2%), 73 left posterior communicating arteries (66.4%) and 18 posterior communicating arteries (16.3%) on both the sides were considered hypoplastic.

**Conclusions:**

In patients without stroke in their history, complete CoW may be present in >60% of the cases. Our diameter data may serve as reference values for the Central-European population.

## 1 Introduction

Although the arterial anastomosis at the base of the brain was described and illustrated before the publication of Cerebri Anatome, it was finally named in honour of Thomas Willis, because he was the one describing the functional role of this collateral system by stating: “…so if by chance one or two should be stopt, there might easily be found another passage instead of them; as for example if the Carotid of one side should be obstructed, then the Vessels of the other side might provide for either Province ….Further, if both the Carotids should be stopt, the offices of each might be supplied through the Vertebrals.” [1]

The circle of Willis (CoW) represents a polygonal anastomotic network located at the base of the brain. Its main role is to provide sufficient collateral blood flow to all regions of the brain and cerebellum, especially in cases of severe large vessel stenosis and occlusions. For a long time, it was believed that ischemic strokes are of haemodynamic origin in approximately one-third of the cases and it was thought that they are caused by severe stenosis or occlusion of the internal carotid artery along with an incomplete intracranial collateral network.

The typical pattern and the variations of the CoW have been assessed extensively in the past few decades in anatomical and radiological studies. Based on these reports, it seems that the “typical pattern” of the textbook polygon can be observed in 4.6–72.2% of the cases [2–4]. Among others, genetic, environmental and haemodynamic factors may explain these variations in the prevalence [5].

The assessment of completeness and the variations of the CoW is still a matter of thorough research because there are data referring to an association between an incomplete circle and ischemic stroke [6], an incomplete circle may worsen patient’s outcome after an ischemic stroke [7] and may increase the risk of intraoperative ischemic events during carotid endarterectomy [8]. Furthermore, observations show that variations of CoW may contribute to the development of cerebral aneurysms [9] or may be associated with white matter disease [10]. It has to be noted that besides morphological features of the circles, the diameter of the collateral vessels also plays an important role in the collateral capacity of the CoW. In the majority of the previous studies, a <1 mm diameter was considered hypoplastic for anterior and posterior communicating arteries [11,12]. However, clinical and mathematical studies indicated that the threshold diameter allowing functional cross-flow through the communicating arteries of the CoW is between 0.4 and 0.6 mm [13–16].

In the present study, we attempted to assess the different anatomical variations of the CoW in a Hungarian non-cerebrovascular cohort. Our second aim was to describe the diameters of the different arterial segments forming the circle and to assess the completeness of the CoW based on the functional diameter threshold.

## 2 Methods

CoWs were removed during the routine autopsy of patients who died at the University of Debrecen hospital facility due to other reasons than a cerebrovascular accident between the period of January 2000 and June 2000. The prerequisite of inclusion was no peripheral vascular disease and/or cerebrovascular accident in the history of the autopsied individuals.

### 2.1 Preparation and removal of the circles

**Figure 1:**
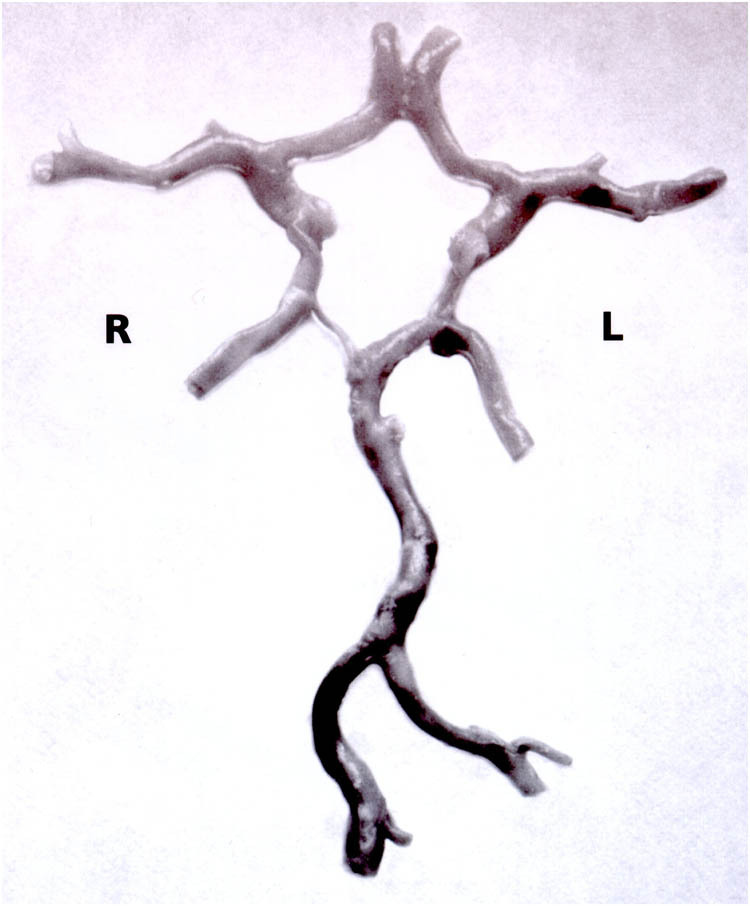
The CoW after removal.

During the routine autopsy, brains were removed from the skull by the pathologists and removal of the circles occurred in the autopsy room on fresh, non-fixed brains. After the evaluation of the pathologist, the removal team started the preparation of the circles from the base of the brain starting from the vertebral and basilar artery. Only the middle-sized arteries of the circles were kept for further measurements, small side branches were removed. By trying for the best possible atraumatic preparation, surgical tweezers and Cooper shear were used. Preparation on the basis of the brain occurred from the posterior to the ventral direction. For the posterior cerebral, middle cerebral and anterior cerebral arteries approximately 2 cm segments after the branching of the corresponding communicating artery were left for later diameter measurements. We tended to remove the arachnoid coverage of the arterial segments from the branches of the circles and as a final step, the side was marked using surgical thread. Figure 1 shows the Willisian circuit after preparation.

As a first step, after the removal of the circles, the structure of the CoW was recorded to document the morphology and eventual anatomical variations. Recording occurred both using photographical and graphical documentation in each case. Based on the structural changes of the CoW we defined the morphological malformations and anatomical variations. The definition for *malformations* was kept for those CoWs where one or more segments of the collaterals were missing so that the circle was unable to fulfil the required collateral function. With *anatomical variations* it was meant that the circle was able to provide collateral supply but either the branching of the artery was different from the classical description or duplications of one or more segments were present.

### 2.2 Measurement of the diameters and lengths of the Willisian segments

**Figure 2:**
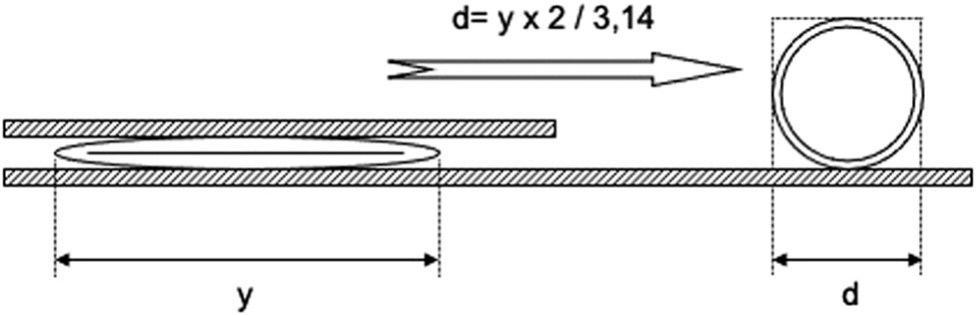
Measurement of the half of the circumference of the arterial segments and calculating the diameter.

After preparation blood was carefully washed out from the CoW with isotonic saline. For measuring the lengths and diameters, the CoW was put on a glass plate. The arteries had been straightened out, the lengths of the PcoAs and A1 segments were measured with a ruler with a millimetre scale and measurements were rounded off to whole millimetres. For the measurement of the length of the AcoA and P1 segments, a transparent 10 cm × 10 cm sheet with a millimetre scale was placed on top of the circle. Under a microscope with a 10× magnification, AcoA and P1 lengths were measured with an approximation of 0.1 mm. For the measurement of the arterial diameters, a second glass plate was clamped on top of the first, with the CoW and the transparent sheet in between. Sufficient force was applied to induce complete obliteration of the vessel lumina but not to flatten the arterial walls. This allowed the application of equal pressure on the entire preparation and thus obliteration of the vessel lumina. The set of glass plates was put under the microscope again. The readings representing half of the arterial circumference were obtained by the measurements under a light microscope. Measurements were taken at proximal, middle and distal sites of the AcoA, A1 segment, PcoA and P1 of the posterior cerebellar artery (PCA) segment and approximated to the nearest 0.1 mm. The narrowest part of each artery was also used for further analysis because we considered that the diameter at this part determined the collateral ability. Assuming the arteries to be circular, their external diameter could be calculated by the formula, diameter = circumference/π. See Figure 2 for demonstration.

### 2.3 Statistical analysis

All row data were analysed using normality test first. Based on this, the diameters of the different vessel segments are expressed in medians and 25–75% confidence intervals or means and standard deviations. Side and gender differences were compared using the appropriate *t*-test.


**Ethical approval:** The research related to human use has complied with all the relevant national regulations, and institutional policies and in accordance with the tenets of the Helsinki Declaration, and has been approved by the Ethics Committee at the University of Debrecen (RKEB-TUKEB 328/1999 OEC).
**Informed consent:** Informed consent has been obtained from representatives of all individuals included in this study.

## 3 Results

Fifty-five males and 55 females with an average age of 70.58 ± 13.2 years were abducted. The cause of death based on autopsy were malignancy (*n* = 34) pneumonia-related respiratory insufficiency (*n* = 26), pulmonary embolism (*n* = 15), chronic decompensated heart insufficiency (*n* = 23), end-stage liver failure (*n* = 7) and intoxication (*n* = 10).

### 3.1 Anatomical malformations

**Figure 3:**
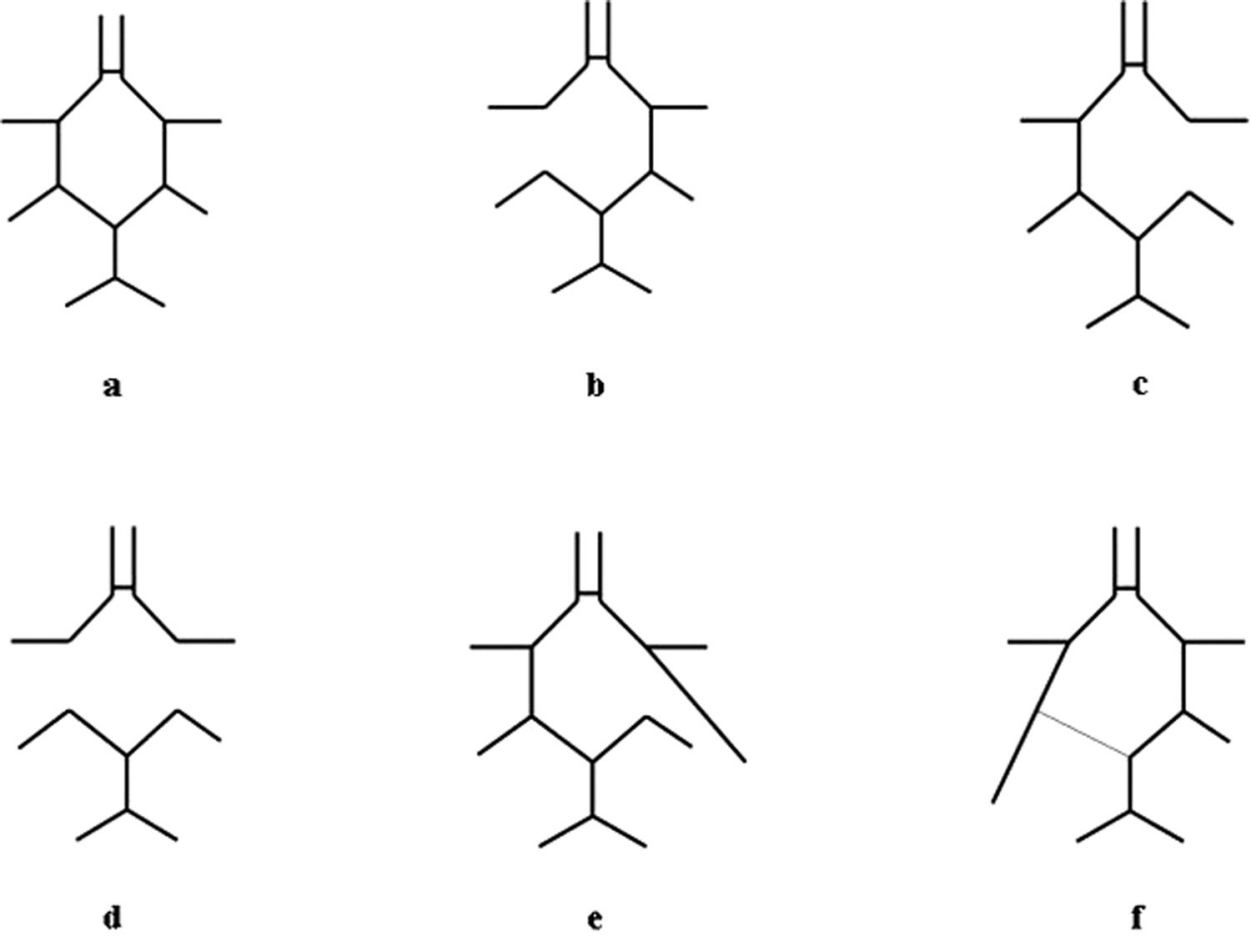
Anatomical malformations of the CoW. The graph demonstrates the normal collateral structure for the sake of comparison (a) and the different malformations found in the cohort (b–f).

An incomplete CoW was found in 25 cases out of the total 110 preparations, corresponding to 22.7%. The distribution of the malformations was as follows (see Figure 3 for better demonstration):

- In 11 cases (10%) posterior communicating artery on the left side was completely missing (Figure 3b).
- In eight cases (7.27%) right posterior communicating artery was missing (Figure 3c).
- In four cases (3.63%) communicating posterior arteries were missing on both the sides (Figure 3d).
- In one case (0.9%) the right anterior communicating artery did not make the circle complete, but rather run independently to the lateral direction (Figure 3e).
- We also considered the circle incomplete in one case (0.9%), where the fetal configuration was found, i.e. the posterior cerebral artery originated from the internal carotid artery and did not make the circle complete. The P1 segment of the posterior cerebral artery in this case was found as a rudimental connective tissue band without a lumen (Figure 3f).

### 3.2 Anatomical variations

**Figure 4:**
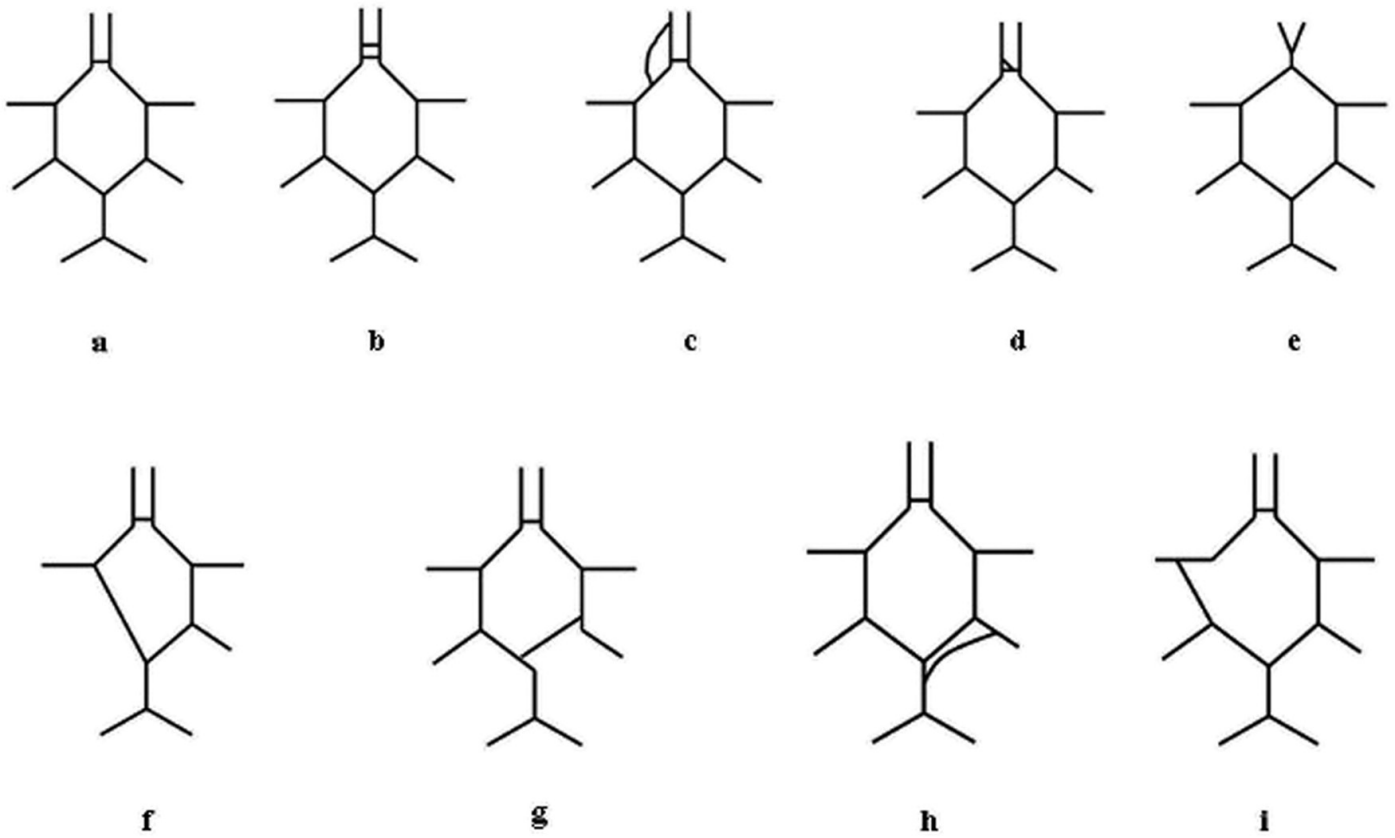
Anatomical variations of the CoW. The graph demonstrates the normal collateral structure for the sake of comparison (a) and the different variations found in the cohort (b–f).

Anatomical variations were found in 14 cases in our cohort, making up 12.7%. The distribution of these variations was as follows (Figure 4)

- In seven cases (6.36%) the communicating artery was duplicated (Figure 4b).
- In one case (0.9%) the A1 segment of the anterior cerebral artery was duplicated (Figure 4c).
- In one case (0.9%) anterior communicating artery showed a Y-shaped appearance (Figure 4d).
- We also found one CoW (0.9%), where the two A1 segments showed a common origin and the A2 segments bifurcated in the later course (Figure 4e).
- In one case (0.9%) the left posterior communicating artery was missing and the posterior cerebral artery was directly connected to the middle cerebral artery (Figure 4f).
- In one circle (0.9%) the P1 segment of the right posterior cerebral artery originated from the left PCA and not from the basilar artery (Figure 4g).
- In one case (0.9%) a duplicated right posterior cerebral artery was found (Figure 4h).
- Finally, in a single case (0.9%) the posterior communicating artery made a connection between the posterior cerebral artery and the distal part of the middle cerebral artery on the left side (Figure 4i).

### 3.3 Diameter measurements on the different segments of the CoW

**Figure 5:**
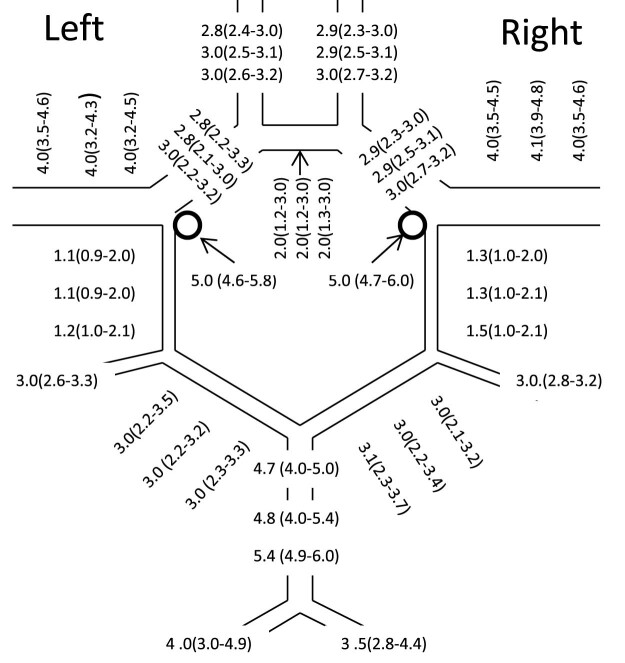
Diameters of different segments of the arteries forming the CoW. Medians and 25–75% IQRs are shown in millimetres.

The results of the three diameter measurements at the different segments of the arteries forming the CoW are demonstrated in Figure 5.

### 3.4 Comparison of side differences of the narrowest part of each arterial segments

In the second step of the analysis, we took the smallest diameter from the three measurements of each segment into account. This was decided because it is conceivable that these parts of the artery determine blood flow through the circle. Additionally, we compared the diameters of paired arteries on the two sides of the CoW to see whether a side predominance exists. The results of this analysis are summarized in Table 1. According to the results of this comparison, the right A1 segment of the anterior cerebral artery and left posterior communicating artery were narrower in our cohort.

**Table 1:** Narrowest diameters (in millimetres) of the different arterial segments and their side-comparison (means ± SDs are shown)

	Right	Left	*p*-Value
Basilar artery	2.73 ± 0.59		—
Internal carotid artery	3.57 ± 0.66	3.31 ± 0.69	0.3
Posterior cerebral artery segment P1	1.64 ± 0.58	1.69 ± 0.55	0.24
Posterior communicating artery	0.80 ± 0.50	0.68 ± 0.52	<0.05
Anterior cerebral artery segment A1	1.48 ± 0.48	1.61 ± 1.27	<0.05
Anterior communicating artery	1.30 ± 0.67		—
Posterior cerebral artery segment P2	1.70 ± 0.70	1.65 ± 0.76	0.29
Middle cerebral artery	2.44 ± 0.41	2.35 ± 0.49	0.08
Anterior cerebral artery segment A2	1.68 ± 0.45	1.67 ± 0.39	0.41

### 3.5 Gender differences in the narrowest diameters

The narrowest diameters were compared among females and males to see whether a gender difference exists. It was found that several arterial segments have significantly lower diameters in females, among them the most pronounced was the basilar artery. Results are shown in Table 2.

**Table 2:** Gender difference of the narrowest segments of the different arteries

	Male	Female	*p*-Value
Basilar artery	2.88 ± 0.52	2.58 ± 0.62	<0.05
Left internal carotid artery	3.35 ± 0.63	3.27 ± 0.76	0.30
Right internal carotid artery	3.37 ± 0.68	3.35 ± 0.66	0.43
Left middle cerebral artery	2.42 ± 0.55	2.30 ± 0.42	0.10
Left anterior cerebral artery A1 segment	1.62 ± 0.51	1.61 ± 0.44	0.46
Left anterior cerebral artery A2 segment	1.68 ± 0.39	1.67 ± 0.40	0.44
Anterior communicating artery	1.46 ± 0.78	1.15 ± 0.51	<0.05
Right middle cerebral artery	2.53 ± 0.45	2.36 ± 0.36	<0.05
Right anterior cerebral artery A1 segment	1.50 ± 0.56	1.47 ± 0.39	0.39
Right anterior cerebral artery A2 segment	1.74 ± 0.51	1.62 ± 0.38	0.09
Right posterior communicating artery	0.79 ± 0.43	0.81 ± 0.58	0.40
Right posterior cerebral artery P1 segment	1.70 ± 0.55	1.57 ± 0.61	0.12
Right posterior cerebral artery P2 segment	1.86 ± 0.55	1.54 ± 0.82	<0.05
Left posterior communicating artery	0.66 ± 0.51	0.70 ± 0.54	0.35
Left posterior cerebral artery P1 segment	1.85 ± 0.48	1.53 ± 0.59	<0.05
Left posterior cerebral artery P2 segment	1.76 ± 0.74	1.54 ± 0.78	0.07

Means ± SDs are shown, values are expressed in millimetres.

### 3.6 Length of the arterial segments

Data on the length of the vessels are summarized in Table 3. There was a gender difference in the two arterial segments. Similar to the diameter data, the most significant differences were observed in the basilar arteries of females and males.

**Table 3:** The length of the different arterial segments in millimetres

	Male	Female	*p*-Value
Basilar artery	31.15 ± 6.51	21.75 ± 5.92	<0.01
Left posterior cerebral artery P1 segment	6.48 ± 3.48	6.29 ± 3.90	0.39
Left posterior communicating artery	12.06 ± 5.71	10.61 ± 6.26	0.1
Left anterior cerebral artery A1 segment	14.10 ± 2.74	12.78 ± 3.18	<0.05
Anterior communicating artery	2.92 ± 2.48	2.64 ± 1.85	0.25
Right posterior cerebral artery P1 segment	14.55 ± 2.44	13.84 ± 4.13	0.14
Right posterior communicating artery	13.35 ± 4.92	12.14 ± 8.52	0.18
Right anterior cerebral artery A1 segment	7.73 ± 4.17	7.06 ± 5.79	0.24

Comparison of the two sexes. Means and standard deviations are shown.

## 4 Discussion

In the present study, we found a 22.7% prevalence of incomplete CoW in our non-cerebrovascular cohort. In 12.7% of the cases, different anatomical variations were described that did not have the potency to hamper collateral ability. In 71 cases (64.6%) a typical configuration of the CoW could be found. Our results are comparable with the description of Fawcett and Blachford who described a 72% rate of normal CoW in ref. [3] and the 52.3% demonstrated by Alpers [17] but significantly different from other descriptions reporting on 11.9% [18], 20.9% [4], 26.8% [19] and 48% [5]. These data demonstrate significant ethnical differences in CoW variations.

From a clinical point of view, there are different reasons for the systematic assessment of the variations of CoW. First, it has been shown that incompleteness and variations of the circle may increase the risk of ischemic strokes by 1.4 times [20] and in patients with severe internal carotid artery occlusions. The Odds ratio of an ischemic stroke in the case of nonfunctional anterior collateral is 7.3, whereas a for nonfunctional posterior collateral pathway it is 3 [6]. Further investigations also proved that incomplete anterior collateral along with incomplete posterior collateral is associated with anterior circulation stroke [21] and posterior communicating artery hypoplasia is associated with the risk of thalamic lacunar lesions [22] even in the absence of severe carotid stenosis. There are observations suggesting that an incomplete CoW may increase the risk of ischemic events after cross-clamping during carotid endarterectomies [8]. Reports suggest a preventive interplay between Willisian and leptomeningeal collaterals during and after revascularization therapies in large vessel occlusions [23].

Different classification schemes were used in the past few decades for assessing the variations of the CoW. These studies included one or more diameter thresholds for arteries [21,24,25], while others divided the CoW to anterior and posterior half-ring [26,27] for assessing completeness. In the present study, we attempted to assess the circles as a whole and in the first step, a circle was considered complete if all segments of the CoW were present, independent of their diameters. In the second step, we used combination of morphological and functional parameters that also included the diameters of the collateral vessels. Thus far, majority of the studies used 1 mm as the threshold diameter for defining of hypoplasia of the collateral vessels [28]. If one uses the classical diameters’ threshold of <1 mm for the definition of hypoplasia, in the present cohort 36 anterior communicating arteries (32.7%), 53 right posterior communicating arteries (48.2%), 73 left posterior communicating arteries (66.4%) and 18 posterior communicating arteries (16.3%) on both sides were considered hypoplastic. However, clinical and mathematical modelling studies indicated that the threshold diameter allowing functional cross-flow through the communicating arteries of the CoW is between 0.4 and 0.6 mm [13–16]. We performed a second analysis that took into consideration that according to the previous clinical and mathematical modelling studies the threshold diameter allowing functional collateral flow is between 0.4 and 0.6 mm [13,14,16], and found that using these threshold diameters five anterior communicating arteries (4.5%), 11 right posterior communicating arteries (10%), 21 left posterior communicating arteries (19.1%) and three posterior communicating arteries on both sides could be considered hypoplastic. Taking into consideration that incompleteness of the CoW is based on the diameters of the communicating arteries, it may be suggested that in further studies threshold diameters gathered from functional and modelling studies should be taken into account.

To our best knowledge, this is the first study that measured vessel diameters forming the CoW at three different points of the arterial segments in fresh, non-fixed autopsy materials. Previous reports used formalin-fixed brains and measured the diameters after ethanol washout [29,30]. In comparison to these data, our diameter values are somewhat underestimated. The main difference between the results may be explained by the shrinkage of the arteries using formalin fixation followed by ethanol treatment that might have affected the elastic properties of the arteries and hence diameter measurements. In a study similar to ours, Kamath also used fresh obduction material for measurements and reported on the comparable diameters of the different Willisian vessels to ours [15]. When comparing diameter data with those obtained from imaging studies, data on diameters of the communicating arteries are scarce, and completeness of the CoW is merely considered if flow in all segments of the circuit can be detectable. In a study by El-Barhoun et al., the normal diameter values of the middle anterior and posterior cerebral arteries were reported 3–2–2 millimetres, respectively, which is somewhat lower than those we observed (4.0–2.9 and 3.0, respectively), but corresponds to our narrowest diameters [31]. Similar to this, Shatri et al. in their MRI study reported similar diameters to our narrowest diameters [32]. This can be explained by taking into consideration that the narrowest part of the vessel determines the blood flow than can be assessed by imaging techniques and diameter measurements can be performed based on this. Table 3 shows a comparative analysis of vessel diameters in autopsy studies.

Only a few studies assessed the gender differences of the Willisian vessels, and the majority could not find any significant differences [33,34]. Using angiography, Shatri et al. reported a significantly larger diameter in males than in females in all segments but the posterior communicating artery [35]. The gender difference warrants further extensive research, especially because there are observations suggesting that the morphology of the CoW may contribute to the risk of aneurysm formation in females [36] (Table 4).

**Table 4:** Diameters of the different segments forming the CoW published in autopsy studies

	Klimek–Piotrowska (means ± SD)	Kamath (means ± SD)	Wasi (means ± SD)	Wijesinghe (means ± SEM)	Present study (means ± SD)
ICAR	3.6 ± 0.8	4.2 ± 0.9	NA	3.0 ± 0.06	3.57 ± 0.66
ICAL	3.6 ± 0.7		NA	3.0 ± 0.07	3.31 ± 0.69
ACAR	2.3 ± 0.6	2.2 ± 0.6	2.3 ± 0.4	1.59 ± 0.04	1.48 ± 0.48
ACAL	2.3 ± 0.5		2.26 ± 0.4	1.57 ± 0.04	1.61 ± 1.27
AComA	1.9 ± 0.7	1.9 ± 0.9	1.85 ± 0.4	1.14 ± 0.07	1.30 ± 0.67
MCAR	NA	NA	NA	2.27 ± 0.04	2.44 ± 0.41
MCAL	NA	NA	NA	2.26 ± 0.04	2.35 ± 0.49
BA	NA	NA	NA	2.72 ± 0.06	2.73 ± 0.59
PCAR	2.2 ± 0.7	2.1 ± 0.7	2.17 ± 0.45	1.6 ± 0.05	1.64 ± 0.58
PCAL	2.3 ± 0.7		2.18 ± 0.7	1.64 ± 0.05	1.69 ± 0.55
PComAR	1.4 ± 0.4	1.5 ± 0.7	1.5 ± 0.2	1.0 ± 0.06	0.80 ± 0.50
PComAL	1.4 ± 0.4		1.5 ± 0.2	0.88 ± 0.05	0.68 ± 0.52

In conclusion, in this descriptive study on non-cerebrovascular individuals, we found that a complete CoW may be present in more than 60% of the cases. As the diameters of the main Willisian collaterals may increase the risk of ischemic strokes in high-risk patients [37], the present data may serve as reference values for the Central-European population when assessing the diameters of the different vessels forming the CoW.
